# Anti-Malignant Effect of Tensile Loading to Adherens Junctions in Cutaneous Squamous Cell Carcinoma Cells

**DOI:** 10.3389/fcell.2021.728383

**Published:** 2021-11-11

**Authors:** Oleg Dobrokhotov, Masaki Sunagawa, Takeru Torii, Shinji Mii, Keiko Kawauchi, Atsushi Enomoto, Masahiro Sokabe, Hiroaki Hirata

**Affiliations:** ^1^ Mechanobiology Laboratory, Nagoya University Graduate School of Medicine, Nagoya, Japan; ^2^ Department of Pathology, Nagoya University Graduate School of Medicine, Nagoya, Japan; ^3^ Division of Surgical Oncology, Department of Surgery, Nagoya University Graduate School of Medicine, Nagoya, Japan; ^4^ Frontiers of Innovative Research in Science and Technology, Konan University, Kobe, Japan

**Keywords:** actomyosin, adherens junction, contact inhibition, mechanosensing, mechanotransduction, epidermoid carcinoma

## Abstract

Actomyosin contractility regulates various cellular processes including proliferation and differentiation while dysregulation of actomyosin activity contributes to cancer development and progression. Previously, we have reported that actomyosin-generated tension at adherens junctions is required for cell density-dependent inhibition of proliferation of normal skin keratinocytes. However, it remains unclear how actomyosin contractility affects the hyperproliferation ability of cutaneous squamous cell carcinoma (cSCC) cells. In this study, we find that actomyosin activity is impaired in cSCC cells both *in vitro* and *in vivo*. External application of tensile loads to adherens junctions by sustained mechanical stretch attenuates the proliferation of cSCC cells, which depends on intact adherens junctions. Forced activation of actomyosin of cSCC cells also inhibits their proliferation in a cell-cell contact-dependent manner. Furthermore, the cell cycle arrest induced by tensile loading to adherens junctions is accompanied by epidermal differentiation in cSCC cells. Our results show that the degree of malignant properties of cSCC cells can be reduced by applying tensile loads to adherens junctions, which implies that the mechanical status of adherens junctions may serve as a novel therapeutic target for cSCC.

## Introduction

The great significance of actomyosin cytoskeleton and actomyosin-generated tension in epithelia development, morphogenesis, and homeostasis is well appreciated. Actomyosin network regulates cell-cell and cell-extracellular matrix (ECM) adhesions, cell division, and delamination, as well as modulates critical regulatory gene networks controlling cell proliferation, apoptosis, stemness, and differentiation (Humphrey et al., 2014; Heer and Martin, 2017; Pinheiro and Bellaïche, 2018). Given the importance of actomyosin in normal epithelial functions, failures in the regulation of the actomyosin cytoskeleton potentially cause various diseases including cancers (Dobrokhotov et al., 2018). Many studies have shown the role of actomyosin in cancer progression; however, these studies have mainly focused on the effect of mechanical forces at the cell-ECM interface rather than cell-cell interaction (Butcher et al., 2009; Provenzano et al., 2009; Yu et al., 2011; Chaudhuri et al., 2018). As most of the solid tumors originate in epithelia in which cells form epithelial sheets (Hanahan and Weinberg, 2011), the mechanical interaction between cells may also influence tumor cell behaviors.

Loss of cell density-dependent inhibition of proliferation (termed contact inhibition of proliferation, CIP) is a characteristic feature of cancer cells (Hanahan and Weinberg, 2011). CIP depends on the formation of the cell-cell adhesion structure, adherens junction (AJ) (McClatchey and Yap, 2012), and dysregulation of AJs is associated with carcinogenesis (Vasioukhin, 2012; Wong et al., 2018). At AJs, the transmembrane protein E-cadherin binds *trans*-homophilically *via* its extracellular domain, which mediates adhesion between the neighboring cells. The cytoplasmic tail of E-cadherin connects to the actomyosin cytoskeleton at AJs through linker proteins, including α- and β-catenins (Mège and Ishiyama, 2017; Bruner and Derksen, 2018). Interestingly, the homophilic binding of E-cadherin *per se* does not inhibit but promotes the proliferation of cells (Liu et al., 2006; Hirata et al., 2017), and actomyosin-generated tension at AJs is essential for CIP in normal human keratinocytes (Hirata et al., 2017). However, the role of tensile forces at AJs in cell proliferation may be context- and cell-type-dependent because it has been reported that tensile loads at AJs induce both promotion and inhibition of proliferation of MDCK cells (Benham-Pyle et al., 2015; Furukawa et al., 2017). In the case of cutaneous squamous cell carcinoma (cSCC), some studies implied the tumorigenic effect of actomyosin contractility (Samuel et al., 2011; García-Mariscal et al., 2018; Carley et al., 2021), while others suggested its tumor-suppressive function (Sugai et al., 1992; McMullan et al., 2003; Strudwick et al., 2015). Notably, neither of the studies directly compared the level of actomyosin contractility in cSCC cells against normal keratinocytes. Hence, it remains unclear whether and how the tensile status at AJs contributes to the malignant proliferation of cSCC cells.

Skin homeostasis depends not only on the regulation of cell proliferation but also on the differentiation of keratinocytes. The epidermis is composed of stratified layers of cells; among them, only cells in the stratum basale, the innermost layer, have the proliferative ability, while cells that lack contact with the basement membrane, form the suprabasal layers and undergo terminal differentiation (Simpson et al., 2011) depending on tension in the nuclear lamina (Carley et al., 2021). In cancer, histological differentiation grade, which assesses how much the cancer cells lose the differentiated phenotype (including expression of differentiation markers and typical morphologies of differentiated cells) of the original tissue cells, is a major prognostic parameter. Less-differentiated cancers generally exhibit more aggressive and drug-resistant phenotypes (Karantza, 2011; Dmello et al., 2019). In the case of cSCC, the low differentiation group shows over three times higher metastatic rate and over two times higher recurrence rate than the high differentiation group (Rowe et al., 1992; Barksdale et al., 1997; Alam and Ratner, 2001). However, it remains unknown whether actomyosin activity affects the differentiation status of cSCC cells.

In this study, we investigate the role of actomyosin contractility in cSCC cell proliferation and differentiation. We find that the overproliferation of cSCC cells is associated with impairment in actomyosin contractility, both *in vivo* and *in vitro*. Exogenous induction of tensile loads at AJs by pharmacological activation of actomyosin or mechanical stretch of confluent cSCC cells attenuated proliferation and induced epidermal differentiation of cSCC cells. Our results suggest that tensile loads at AJs have an anti-malignant effect in cSCC.

## Results

### Overproliferation of cSCC Cells Concurs with Impaired Actomyosin Contractility

**FIGURE 1:**
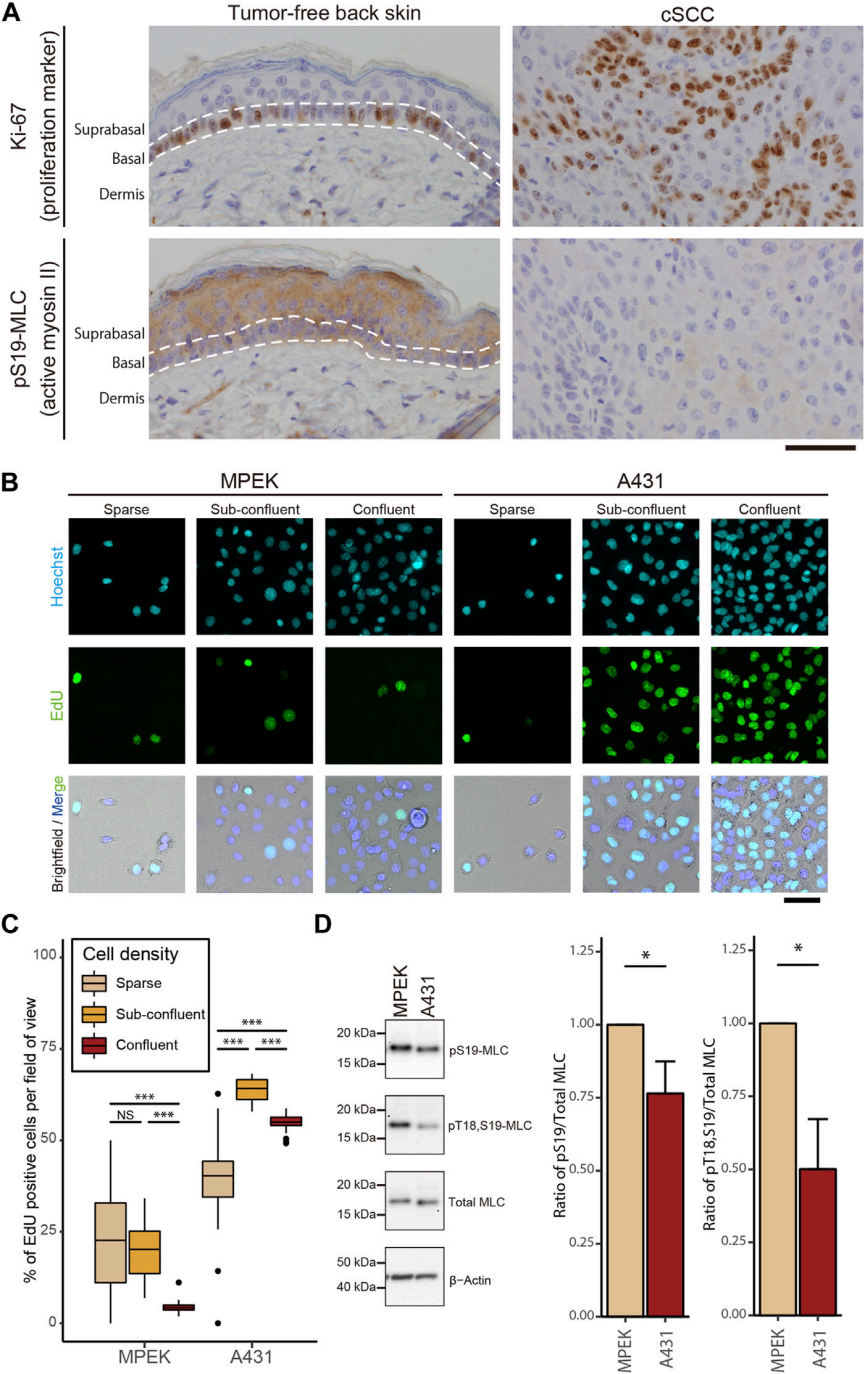
Impairment of actomyosin contractility is associated with hyperproliferation of cSCC cells both *in vitro* and *in vivo*. **(A)** DMBA/TPA-treated tumor-free mouse back skin and cSCC were subjected to IHC staining for Ki-67 and pS19-MLC. Scale bar, 50 μm. **(B)** Fluorescence microscopy analysis of EdU incorporation (green) in MPEK and A431 cells under sparse, sub-confluent, and confluent conditions. Under the sparse condition, only sequestered cells lacking any cell-cell contact were used for the analyses. Cell nuclei were labeled with Hoechst 33342 (cyan). Scale bar, 50 μm. **(C)** Percentages of EdU-positive nuclei in MPEK and A431 cells at different cell densities. Box-and-whisker plots show the median, the interquartile range, and the tenth and ninetieth percentiles. *n* = 40 fields of view from two independent experiments (>1500 cells per condition) were pooled for the analysis. ***p < 0.001; NS, *p* > 0.05. **(D)** Whole-cell lysates of confluent MPEK and A431 cells were immunoblotted for pS19-MLC, Thr18-and Ser19-doublephosphorylated MLC (pT18,S19-MLC), total MLC, and β-actin. Bar graphs show the quantification of the densitometric ratio of pS19-MLC or pT18,S19-MLC against total MLC. Values were normalized with the mean values in MPEK cells. Each bar represents mean ± SD from three independent experiments. *p < 0.05.

We first examined actomyosin contractility in cSCC *in vivo* by using mouse skin tumors that were induced chemically by application of 9,10-dimethyl-1,2-benzanthracene (DMBA) followed by tetradecanoyl-phorbol acetate (TPA), which causes mutations that are highly similar to somatic mutations in human cSCC (Nassar et al., 2015). Samples of the tumor-free back skin and DMBA/TPA-induced skin tumors from wild-type FVB/N mice were immunohistochemically (IHC) stained for Ki-67, a cell proliferation marker (Gerdes et al., 1984), and myosin regulatory light chain (MLC) phosphorylated at Ser19 site (pS19-MLC), as the pS19-MLC level reflects the contractile activity of actomyosin (Riento and Ridley, 2003) (Figure 1A), wherein smooth and skeletal muscle cells were used as the internal positive control for pS19-MLC staining (Supplementary Figures S1A,B). Since cSCC cells are deemed to originate from the quiescent suprabasal keratinocytes (Ratushny et al., 2012), we compared cSCC cells with the normal keratinocytes of suprabasal layers. As expected, the tumor-free skin showed typical stratification of cells and Ki-67-positive cells were not detected in suprabasal layers, i.e., keratinocytes in the suprabasal layers were cell cycle-arrested (Figure 1A). By contrast, skin tumors did not show a layered organization, and Ki-67 positive cells were distributed throughout the tumors. The higher ability of cell proliferation in the skin tumors concurred with lower actomyosin contractility in comparison to the suprabasal layers of the healthy epidermis (Figure 1A). We further noted that, in the papilloma tissue, which is a benign tumor that represents an early stage of cancerogenesis and may progress to cSCC (Neagu et al., 2016), MLC phosphorylation and Ki-67 expression had an apparent negative correlation. A high level of pS19-MLC in the superficial region coincided with almost exclusively Ki-67-negative cells, while in the deeper regions MLC phosphorylation was largely reduced and Ki-67-positive cells were abundant (Supplementary Figure S1C). These results demonstrate that the high proliferation ability is closely associated with the low actomyosin activity throughout normal keratinocytes, papilloma, and cSCC cells *in vivo*, and suggest that the reduction in MLC phosphorylation first occurs at a relatively early stage of skin cancer development.

To recapitulate our *in vivo* findings in a cell culture model, we used the cSCC cell line, A431, commonly used for skin cancer studies. As expected and in contrast to primary epidermal keratinocytes (MPEK), A431 cancer cells showed impairment in CIP; even at the confluent cell density, A431 cells continued proliferating (Figures 1B,C). The proliferation of A431 cells was greater at the confluent cell density than that for sequestered cells lacking cell-cell contact, with the maximum reached at the sub-confluent cell density (Figure 1C). Following the assessment of proliferation, levels of MLC phosphorylation were evaluated by western blotting (WB). Consistent with the IHC results *in vivo*, A431 cSCC cells showed a lower level of MLC phosphorylation at both Thr18 and Ser19 residues than normal MPEK keratinocytes, while total MLC expression stayed unaltered (Figure 1D). Taken together, our results reveal that cSCC cells with a high proliferation ability have a reduced level of actomyosin contractility in comparison to normal keratinocytes both *in vivo* and *in vitro*.

### Both Activation of Actomyosin Contractility and Mechanical Stretch Inhibit Cell Cycle Progression in cSCC Cells

**FIGURE 2:**
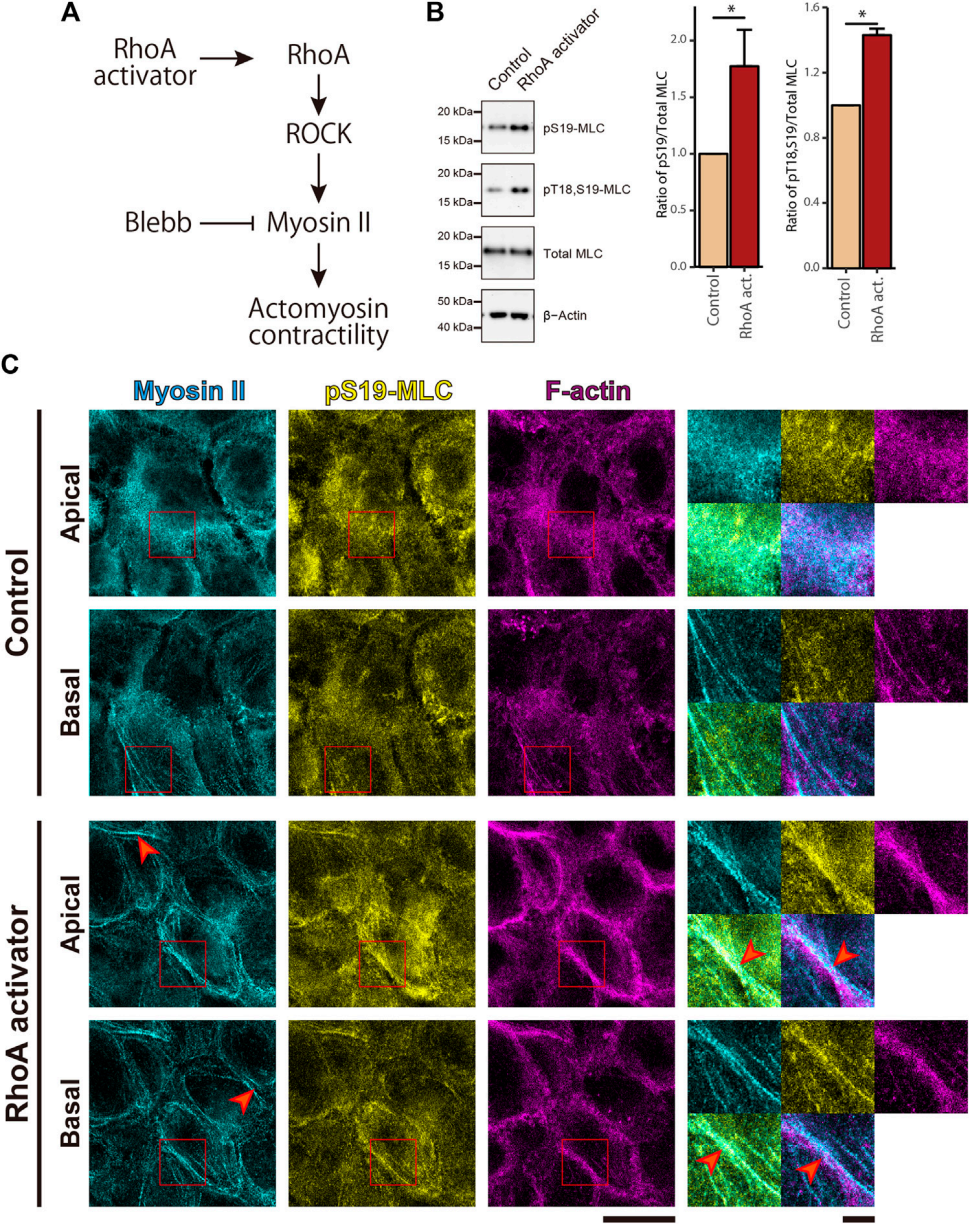
RhoA activation induces MLC phosphorylation and enrichment of actomyosin fibers at the cell-cell junctional regions in cSCC cells. **(A)** The RhoA-ROCK-myosin II signaling cascade for actomyosin activation. **(B)** Whole-cell lysates of control A431 cells and A431 cells treated with 5 μg/ml RhoA activator (RhoA act.) were immunoblotted for pS19-MLC, pT18,S19-MLC, total MLC, and β-actin. Bar graphs show the quantification of the densitometric ratio of pS19-MLC or pT18,S19-MLC against total MLC. Values were normalized with the mean values in control cells. Each bar represents mean ± SD from three independent experiments. *p < 0.05. **(C)** Confluent A431 cells were treated as in **(B)** and stained for non-muscle myosin IIA (cyan), pS19-MLC (yellow), and F-actin (magenta). Apical and basal focal planes of the cells are shown. Magnified images of the boxed regions are also shown. Red arrowheads indicate accumulated actomyosin at the lateral part of the cell cortex in the RhoA activator-treated cells. Scale bars, 5 μm for magnified images, and 20 μm for others.

Given that the high ability of cell proliferation coincides with low actomyosin contractility in cSCC cells, it is natural to ask whether the low level of actomyosin contractility contributes to the overproliferation of cSCC cells. To address this question, we activated the small GTPase RhoA in the RhoA-ROCK-myosin II signaling cascade by treating cells with the membrane-permeable derivative of the bacterial cytotoxic necrotizing factor which selectively deamidates Gln63 of RhoA to constitutively activate RhoA (Flatau et al., 1997; Schmidt et al., 1997) (Figure 2A). Upon RhoA activation in confluent A431 cells, we observed elevated actomyosin contractility as revealed by an increase in Thr18 and Ser19 phosphorylation of MLC (Figure 2B). Activation of actomyosin potentially elevates the tensile status not only at AJs but also at focal adhesions (FAs), which may facilitate autophosphorylation of focal adhesion kinase (FAK) and thus promote cell proliferation (Schwartz and Assoian, 2001; Wang et al., 2005; Pasapera et al., 2010; Hirata et al., 2015). To discriminate actomyosin activities at these distinct adhesion classes, we conducted an immunofluorescence (IF) analysis of actomyosin distribution in the cells. Interestingly, in non-treated control samples, actomyosin was rather dispersed with only few distinct filaments at the basal part of the cortex (Figure 2C). By contrast, RhoA activation induced formation of prominent actomyosin fibers and their accumulation along the lateral membrane at cell-cell boundaries (Figure 2C, red arrowheads). However, this treatment did not apparently promote formation of stress fibers at the basal cortex (Figure 2C).

**FIGURE 3:**
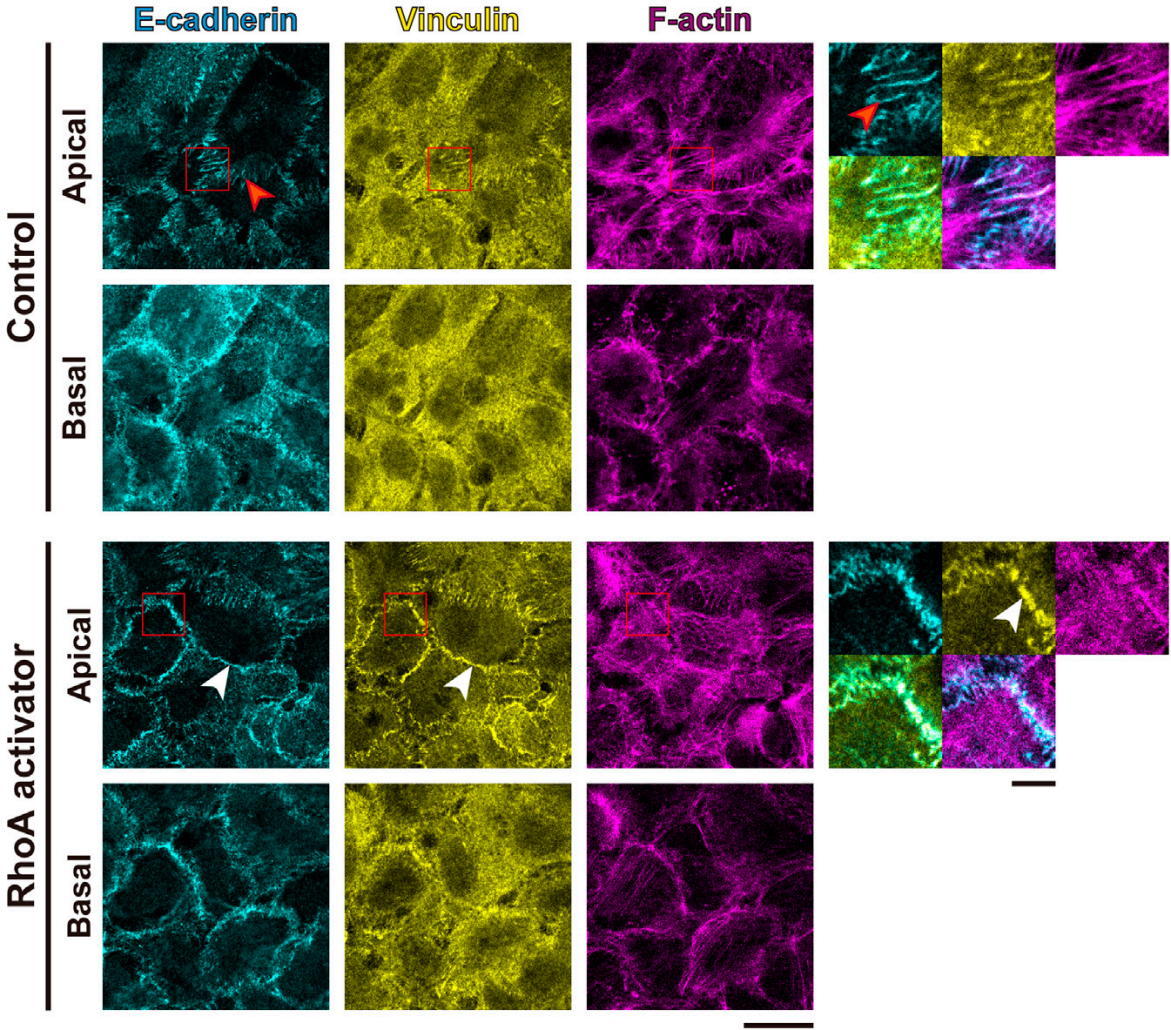
RhoA activation increases tensile loads at adherens junctions but not focal adhesions in cSCC cells. Confluent A431 cells were treated with or without 5 μg/ml RhoA activator (RhoA act.) and stained for E-cadherin (cyan), F-actin (magenta), and vinculin (yellow). Apical and basal focal planes of the cells are shown. Magnified images of the boxed regions are also shown. The red arrowheads indicate zipper-like AJs, and the white arrowheads indicate continuous AJs. Scale bars, 5 μm for magnified images, and 20 μm for others.

To further evaluate the tensile status at cell adhesion structures, we examined the intracellular distribution of vinculin, because vinculin localizes to AJs and FAs when these adhesive structures suffer tensile force (Yonemura et al., 2010; Hirata et al., 2014; Bays and DeMali, 2017). Consistent with the impairment in MLC phosphorylation, in the non-treated confluent A431 cells, vinculin was mainly cytoplasmic with only occasional localization at AJs and without noticeable localization at FAs (Figure 3). However, treatment with the RhoA activator induced vinculin accumulation at AJs (Figure 3). Even though a faint vinculin-positive FAs could be observed in some RhoA activator-treated cells (Supplementary Figure S2A), in majority of the cells vinculin accumulation at FAs was not detected. Similar to vinculin, zyxin (another force-responsive marker of FAs; Hirata et al., 2008; Schiller et al., 2011) also showed only slight accumulation at FAs in small subpopulation of RhoA activator-treated cells (Supplementary Figure S2A). In contrast to A431 cells, C2C12 myoblasts, which were used as a positive control, showed obvious localization of vinculin and zyxin at FAs (Supplementary Figure S2B). In line with these observations, RhoA activation in confluent A431 cells did not altered phosphorylation at Tyr925 site of FAK (Supplementary Figure S3). Taken together, these results suggest that RhoA activation significantly increased tensile loads at the AJs but not at the FAs in confluent A431 cells, which is consistent with the previous finding that actomyosin-generated tension is differentially regulated in distinct subcellular compartments (Gayrard et al., 2018; Déjardin et al., 2020). Notably, while only zipper-like AJs connected to the tips of radial actin bundles were observed in apical portions of control A431 cells (Figure 3, red arrowheads), treatment with the RhoA activator caused the formation of continuous lines of E-cadherin staining associated with the cortical actin and vinculin (Figure 3, white arrowheads), which was reminiscent of the development of honeycomb lattice of continuous AJs in differentiating keratinocytes (Vaezi et al., 2002). Consistently, the length of continuous vinculin-positive AJs was significantly higher under the RhoA activator treatment, in comparison to the control (Supplementary Figures S4A,B).

**FIGURE 4:**
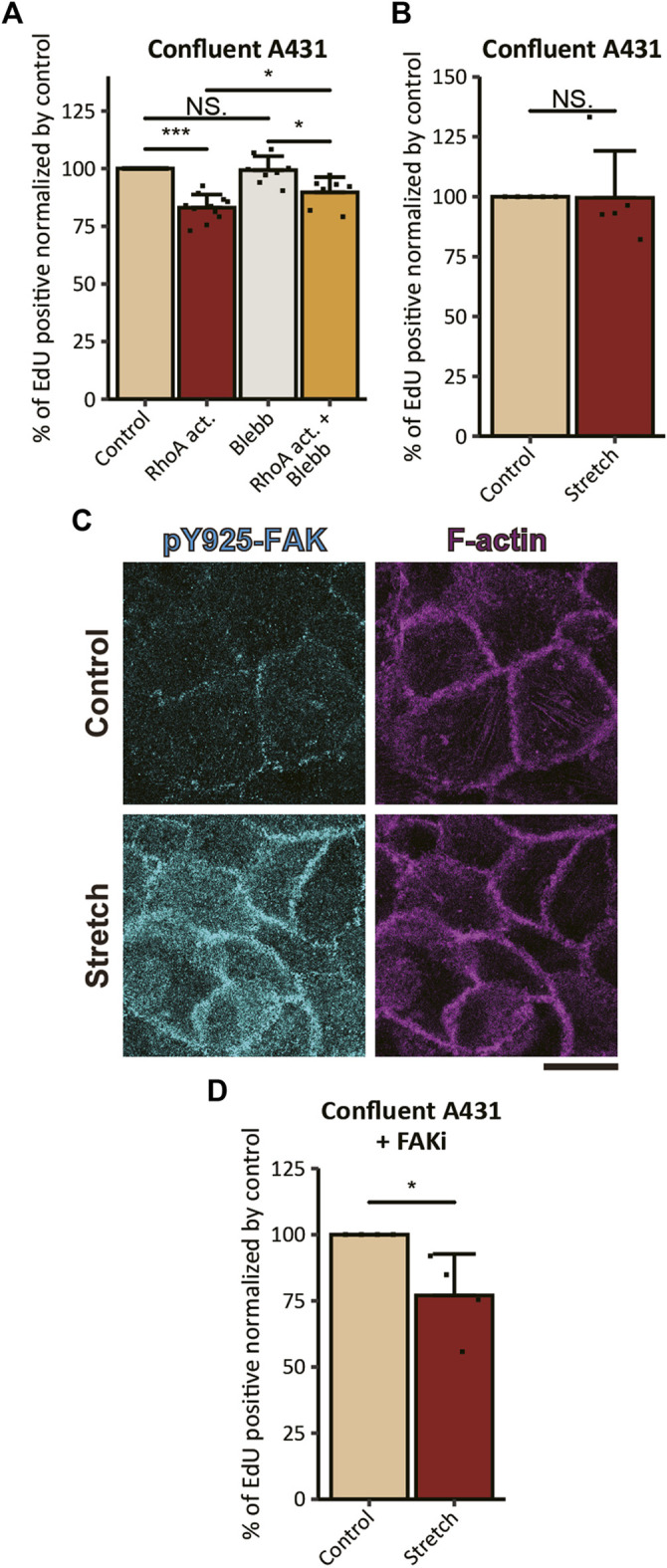
Actomyosin activation and mechanical stretch inhibit the proliferation of cSCC cells. **(A)** Ratios of EdU-positive nuclei in confluent A431 cells treated with 5 μg/ml RhoA activator (RhoA act.), 100 μM *p*-aminoblebbistatin (Blebb) or combination of both drugs (RhoA act. + Blebb) for 6 h followed by incubation with EdU for 2 h in the presence of the same compounds. Values were normalized with the mean value in control cells. Each bar represents mean ± SD. *n* ≥ 8 independent experiments. *p < 0.05; ***p < 0.001; NS, *p* > 0.05. **(B)** Ratios of EdU-positive nuclei in confluent A431 cells without FAKi treatment under static (Control) and 20% UPS (Stretch) conditions. Values were normalized with the mean value in the static control. Each bar represents mean ± SD. *n* = 5 independent experiments. NS, *p* > 0.05. **(C)** Non-treated confluent A431 cells under static (Control) and 20% UPS (Stretch) conditions were stained for pY925-FAK (cyan) and F-actin (magenta). Basal focal planes of the cells are shown. Scale bars, 20 μm. **(D)** Ratios of EdU-positive nuclei in confluent A431 cells with FAKi treatment under static (Control) and 20% UPS (Stretch) conditions. Values were normalized with the mean value in the static control. Each bar represents mean ± SD. *n* = 4 independent experiments. *p < 0.05; NS, *p* > 0.05.

Next, we assessed the effect of RhoA activation on cell cycle progression. We found that the proliferation of A431 cells was inhibited by 6-h treatment with the RhoA activator, as revealed by a reduction in the ratio of EdU positive cells in comparison to the non-treated control (Figure 4A). The RhoA-induced inhibitory effect on cell proliferation was diminished by combined treatment with the direct inhibitor of myosin II ATPase activity, *p*-aminoblebbistatin (hereafter Blebb; Várkuti et al., 2016), indicating that RhoA activation attenuates proliferation of cSCC cells *via* activation of actomyosin contractility. However, since the Blebb treatment failed to fully abrogate the effect of RhoA activation (Figure 4A), some additional myosin II-independent mechanism(s) might also be involved in RhoA-induced inhibition of cell proliferation. Notably, Blebb treatment alone did not have any statistically significant effect on the proliferation of confluent A431 cSCC cells (Figure 4A), whereas the same treatment led to the promotion of cell proliferation in normal keratinocytes under the confluent condition (Hirata et al., 2017). These opposing results are likely to be caused by the difference in endogenous levels of actomyosin contractility in these two cell types; in contrast to the case of normal keratinocytes with the high actomyosin activity, the low level of actomyosin contractility in cSCC cells would abrogate additional effects of myosin II inhibition on cell proliferation, whilst the increase in actomyosin contractility upon RhoA activation would sensitize cSCC cells to Blebb treatment. Of note, when duration of treatment was extended to 24 h, the proliferation inhibitory effect of RhoA activation was further enhanced (Supplementary Figure S5A). However, 24-h treatment with Blebb caused nuclear fragmentation in a number of cells (Supplementary Figure S5B), which was consistent with the previous report showing that long-term (>15 h) treatment with Blebb had a cytotoxic effect in keratinocytes (Hirata et al., 2017). Hence, 6-h treatment duration was exploited in further experiments.

While RhoA activation upregulates actomyosin contractility through the RhoA-ROCK-myosin II signaling cascade, ROCK is not the sole effector of RhoA (Wheeler and Ridley, 2004) and MLC is not the sole substrate of ROCK (Riento and Ridley, 2003). Hence, RhoA activation potentially brings multiple consequences apart from the induction of actomyosin contractility, which might be reflected by the observation that the proliferation-inhibitory effect of RhoA on cSCC cells was not completely abrogated by Blebb treatment, as discussed above. Another way to induce tension at AJs is a mechanical planar stretch of cell culture (Borghi et al., 2012). However, the application of 20% uniaxial planar stretch (UPS) to the cells seeded on the flexible substrate neither inhibited nor promoted the proliferation of confluent A431 cells (Figure 4B). It is known that substrate stretch increases tension not only at the AJs but also at cell-ECM adhesions (Hirata et al., 2014; Sun et al., 2016; Sethi et al., 2017; Andersen et al., 2018), which might promote cell proliferation through the FAK signaling (Schwartz and Assoian, 2001; Wang et al., 2005). Therefore, the result can be explained by the counteracting effect of tensile loading to the AJs and FAs on the proliferation of stretched A431 cells. Consistently with this assumption, UPS application to confluent A431 cells induced Tyr925 phosphorylation of FAK (Figure 4C) which is known to promote cell proliferation through Ras/MAPK signaling (Schlaepfer et al., 1994). To exclude contribution of the cell-ECM tension-dependent mechanism to stretch-induced responses in cell proliferation, we pre-treated confluent A431 cells with the FAK inhibitor PF-573228 (hereafter FAKi; Slack-Davis et al., 2007). In contrast to non-treated cells, cells treated with FAKi showed a significant reduction in EdU incorporation in response to the application of UPS (Figure 4D). These results suggest that exogenous tensile loading to AJs has an inhibitory effect on the proliferation of cSCC cells, however, UPS application also induces FAK activation that counteracts this inhibitory effect.

### Adherens Junctions are Required for Tensile Loading-Induced Inhibition of the Proliferation in cSCC Cells

**FIGURE 5:**
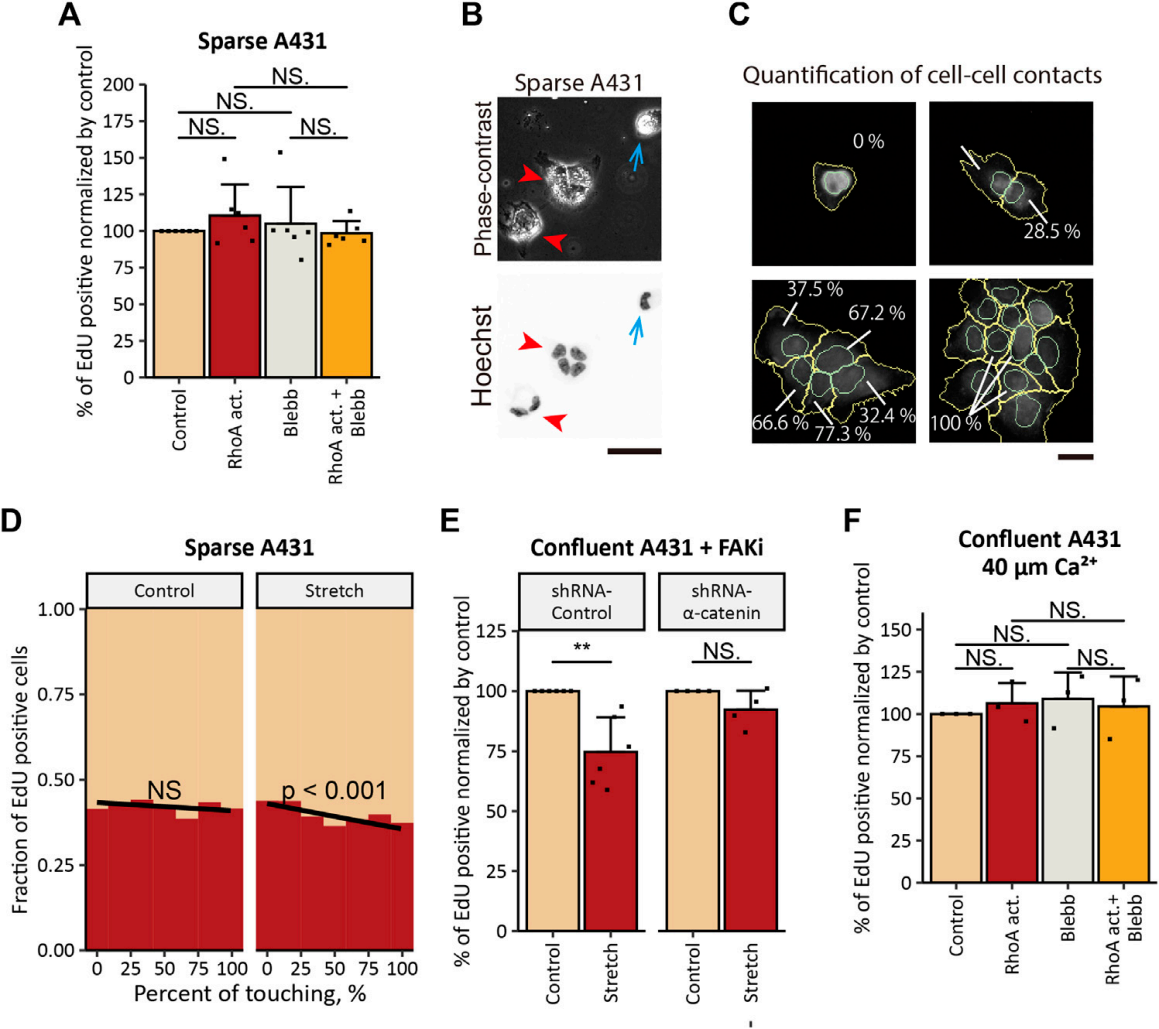
Tensile loading-induced inhibition of the proliferation depends on the intact AJs in cSCC cells. **(A)** Ratios of EdU-positive nuclei in sparse A431 cells treated with 5 μg/ml RhoA activator (RhoA act.), 100 μM *p*-aminoblebbistatin (Blebb) or combination of both drugs (RhoA act. + Blebb) for 6 h followed by incubation with EdU for 2 h in the presence of the same compounds. Sequestered cells lacking any cell-cell contact were used for the analyses. Values were normalized with the mean value in control cells. Each bar represents mean ± SD. *n* = 6 independent experiments (>900 cells each). NS, *p* > 0.05. **(B)** Phase-contrast and fluorescence images of A431 cells seeded at a low cell density. Cell nuclei were labeled with Hoechst 33342. Blue arrows indicate sequestered cell, and red arrowheads indicate cell clusters. Scale bar, 50 μm. **(C)** Graphical representation of the quantification of cell-cell contact. In cells stained for nuclei (with Hoechst 33342) and β-catenin, nuclei (cyan) and whole-cells (yellow) were outlined, and the percentage of the cell perimeter contacting to neighboring cells was quantified using CellProfiler software. Scale bar, 50 μm. **(D)** Under 20% UPS (Stretch, **right panel**), but not under the static condition (Control, **left panel**), the fraction of A431 cells that transited into S-phase decreased with the increase in the percentage of the cell perimeter that was in direct contact with neighboring cells. Results of logistic regression fitting are presented as black lines with the corresponding *p*-values (NS, *p* > 0.05). Results of four independent experiments (>800 cells each) were pooled for the analysis. **(E)** Ratios of EdU-positive nuclei in FAKi-treated A431 cells expressing control-shRNA or α-catenin-shRNA under static (Control) and 20% UPS (Stretch) conditions. Values were normalized with the mean values in static controls. Each bar represents mean ± SD. *n* ≥ 4 independent experiments. **p < 0.01; NS, *p* > 0.05. **(F)** Ratios of EdU-positive nuclei in confluent A431 cells treated as in **(A)** under the low-Ca^2+^ condition. Values were normalized with the mean value in control cells. Each bar represents mean ± SD. *n* = 3 independent experiments. NS, *p* > 0.05.

In our previous study, actomyosin tension specifically at AJs was shown to inhibit the proliferation of normal keratinocytes (Hirata et al., 2017). Thus, we sought to examine the involvement of AJs in the mechanically induced inhibition of proliferation in A431 cells. Cells that lack cell-cell contact *a priori* do not form AJ complexes. Therefore, we evaluated the effect of RhoA activation on the proliferation of A431 cells under the sparse condition. In contrast to the case of confluent A431 cells, the treatment of sequestered A431 cells with the RhoA activator did not show any statistically significant effect on cell proliferation (Figure 5A), which was associated with no apparent promotion of vinculin and zyxin accumulation at FAs (Supplementary Figure S2C).

Additional evidence for the role of the tension at AJs in inhibition of cSCC cell proliferation was obtained by UPS application to A431 cells seeded at a low cell density. As A431 cells do not proceed epithelial-mesenchymal transition and retain an epithelial phenotype (Garnier et al., 2012), they tended to form cell clusters (Figure 5B, red arrowheads) and only some subpopulation of cells stayed secluded (Figure 5B, blue arrows). Using logistic regression analyses, we assessed whether the extent of cell-cell contact (measured as the percentage of the cell perimeter in direct contact with neighboring cells) (Figure 5C) would affect cell proliferation under UPS. We found that cells with larger cell-cell contact regions showed a significantly lower probability of S-phase entry under UPS but not under the non-stretched control condition (Figure 5D). Taken together these results indicate that cell-cell contacts are indispensable for the tensile loading-induced inhibition of the proliferation in cSCC cells.

Another way to examine the role of tension at AJs is to interfere with the force transmission from the actin cytoskeleton to AJs. α-Catenin, one of the major components of AJs, is essential for the mechanical coupling of the actomyosin network to AJs, and the loss of α-catenin functionally impairs AJs (Mège and Ishiyama, 2017) and prevents transmission of actomyosin-generated forces to AJs (Thomas et al., 2013; Barry et al., 2014; Buckley et al., 2014). Accordingly, we stably knocked down (KD) α-catenin expression in A431 cells by the use of shRNA (Supplementary Figures S6A,B). The proliferation inhibitory effect of UPS in confluent A431 cells under FAK inhibition was eliminated by α-catenin KD (Figure 5E), indicating involvement of intact AJs in UPS-induced inhibition of A431 cell proliferation. However, treatment with the RhoA activator caused a significant decrease in cell proliferation in both cells expressing non-targeting shRNA and α-catenin-KD cells cultured at confluent densities, and a decrease in cell proliferation upon RhoA activation was abrogated by Blebb treatment (Supplementary Figure S6C). Potentially, the residual expression of α-catenin in α-catenin-KD cells was sufficient to form immature AJs (Supplementary Figure S6D, red arrowheads), which might trigger proliferation inhibitory signaling downstream of RhoA activation. On the other hand, in contrast to sturdy apical AJs formed in naïve A431 cells (Supplementary Figure S6D, yellow arrowheads), these immature AJs would not be stable enough to withstand more acute mechanical stretch. Such a touchy nature of AJs in α-catenin-KD cells might underlie the phenomenon that α-catenin KD eliminated the proliferation inhibitory effect of mechanical stretch but not the effect of RhoA activation.

To further investigate the role of tension at AJs, we disassembled AJs by depleting extracellular Ca^2+^ from the cell culture medium as Ca^2+^ is required for intercellular homophilic interaction of E-cadherin (Lewis et al., 1994; Koch et al., 1997; Kim et al., 2011). Keratinocytes do not form AJs in media containing 30 μM of Ca^2+^, while AJs rapidly form when Ca^2+^ concentration is raised to 1 mM (Lewis et al., 1994). Consistently with the previous reports, we observed that in the low-Ca^2+^ medium (ca. 40 μM of Ca^2+^) A431 cells lacked apparent AJs (Supplementary Figure S7). Under the low-Ca^2+^ condition, the treatment of A431 cells with the RhoA activator did not show an inhibitory effect on the proliferation of A431 cells (Figure 5F). Taken together, the results obtained for A431 cells show that actomyosin contractility and mechanical stretch inhibit cSCC cell proliferation in an AJ-dependent manner.

### RhoA-Induced Inhibition of cSCC Cell Proliferation Accompanies Promotion of Keratinocytic Differentiation

**FIGURE 6:**
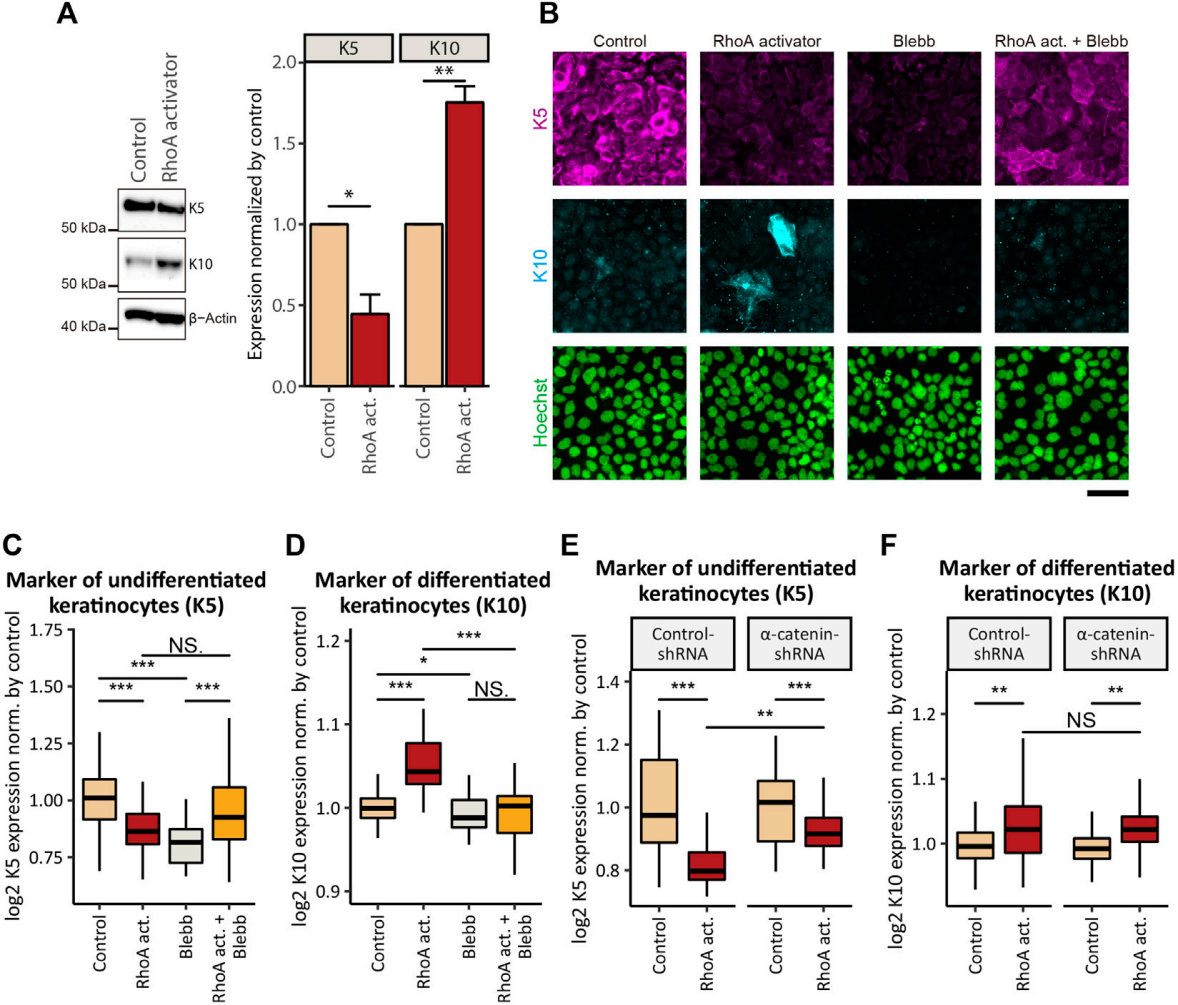
Activation of actomyosin contractility induces epidermal differentiation of cSCC cells. **(A)** Whole-cell lysates of confluent A431 cells treated with or without 5 μg/ml RhoA activator (RhoA act.) for 24 h were immunoblotted for K5, K10, and β-actin. Bar graphs show the result of the densitometric quantification of K5 and K10. Values were normalized with the mean values in control cells. Each bar represents mean ± SD. *n* = 3 independent experiments. *p < 0.05; **p < 0.01. **(B)** Confluent A431 cells treated with 5 μg/ml RhoA activator (RhoA act.), 100 μM *p*-aminoblebbistatin (Blebb) or combination of both drugs (RhoA act. + Blebb) for 24 h were stained for markers of basal (undifferentiated, K5, magenta) and suprabasal (differentiated, K10, cyan) keratinocytes. Cell nuclei were labeled with Hoechst 33342 (green). Scale bar, 50 μm. **(C, D)** Quantification of **(B)**. Fluorescence intensities of K5 **(C)** and K10 **(D)** in confluent A431 cells treated with indicated compounds for 24 h. Values were normalized with the mean values in control cells. Box-and-whisker plots show the median, the interquartile range, and the tenth and ninetieth percentiles. *n* = 60 fields of view from three independent experiments were pooled for the analysis. *p < 0.05; ***p < 0.001; NS, *p* > 0.05. **(E, F)** Quantification of fluorescence intensities of K5 **(E)** and K10 **(F)** in confluent A431 cells expressing control-shRNA or α-catenin-shRNA treated with indicated compounds for 24 h. Values were normalized with the mean values in non-treated controls. Box-and-whisker plots show the median, the interquartile range, and the tenth and ninetieth percentiles. *n* = 60 fields of view from three independent experiments were pooled for the analysis. **p < 0.01; ***p < 0.001; NS, *p* > 0.05.

Considering that proliferation and differentiation are tightly coordinated in normal keratinocytes (Simpson et al., 2011), we next asked whether RhoA-induced inhibition of cSCC cell proliferation was associated with an alteration in the cell differentiation status. Six-hour treatment with the RhoA activator (under the same conditions as for the EdU incorporation assay) did not significantly affect the expression of markers for basal undifferentiated keratinocytes-keratin-5 (K5) and differentiated keratinocytes-keratin-10 (K10) (data not shown). However, when the treatment duration was extended to 24 h, RhoA activation led to a significant decrease in the expression of K5 and an increase in the expression of K10 (Figures 6A–D). Co-treatment with Blebb eliminated the RhoA-induced elevation in K10 expression (Figures 6B,D), indicating that the expression of K10 depends on the actomyosin activity. By contrast, the same co-treatment did not show a statistically significant increase in the K5 expression (*p* = 0.094) in comparison to the treatment with the RhoA activator alone (Figure 6C). However, the ratio of cells expressing high levels of K5 was notably increased by co-treatment with Blebb (the upper quartile of the K5 expression in the cells treated with the combination of the RhoA activator and Blebb was approximately equal to the 90th percentile of K5 expression in cells treated with the RhoA activator alone; Figure 6C). Hence, K5 expression may also be affected by actomyosin-dependent mechanism(s). While treatment with Blebb alone reduced expression of both K5 and K10 (Figures 6C,D), this might be potentially due to the cytotoxic effect of long-term Blebb treatment, as discussed above. Taken together, our results suggest that activation of actomyosin contractility not only inhibits proliferation but also promotes keratinocytic differentiation in cSCC cells.

Next, we assessed the involvement of AJs in the actomyosin-regulated differentiation process. shRNA-mediated KD of α-catenin in A431 cells significantly attenuated the RhoA-induced inhibition of K5 expression in comparison to the cells expressing control-shRNA (Figure 6E), whereas the effect of RhoA activation on K10 expression was not significantly altered by α-catenin KD (Figure 6F). These results suggest that promotion of differentiation of cSCC cells downstream of actomyosin activation depends, at least partially, on intact AJs, while distinct differentiation markers may be influenced by different effectors downstream of RhoA.

## Discussion

The role of actomyosin contractility in cancer cell proliferation has not been well understood. While we have previously reported that actomyosin-based tension at AJs is required for CIP in normal keratinocytes (Hirata et al., 2017), we find in this study that cSCC cells having a defect in CIP exhibit a low level of MLC phosphorylation, which should cause reduced actomyosin contractility. Furthermore, we provide evidence that the proliferation of cSCC cells is inhibited by pharmacologically (i.e., RhoA activation) or mechanically (i.e., UPS application) induced tensile loads at AJs. Importantly, actomyosin activation in cSCC cells not only inhibits their proliferation but also promotes epidermal differentiation in an AJ-dependent manner. Our results suggest that tension at AJs acts as an anti-malignant factor in cSCC.

Our finding that actomyosin contractility inhibits cell cycle progression in cSCC is in line with some of the previous reports showing that RhoA and ROCK are required for keratinocyte differentiation, while ROCK inhibition promotes keratinocyte proliferation (Sugai et al., 1992; McMullan et al., 2003; Strudwick et al., 2015). Nevertheless, other reports imply pro-proliferative and tumorigenic effects of actomyosin contractility in skin keratinocytes (Samuel et al., 2011; García-Mariscal et al., 2018). It is of note that in contrast to the present study, the previous *in vitro* study (Samuel et al., 2011; García-Mariscal et al., 2018) showing the proliferation-promoting effect of actomyosin activity in primary keratinocytes employed low extracellular Ca^2+^ conditions (at concentrations below 20 μM) for experiments. AJ formation would be abrogated under these low Ca^2+^ conditions, and, therefore, activation of actomyosin might not result in AJ-dependent inhibition of cell proliferation. Since the extracellular Ca^2+^ concentration in the epidermis is normally maintained in the range of 1.1–1.4 mM (Breitwieser, 2008), we believe that the condition used in this study (ca. 2.2 mM of Ca^2+^) is physiologically relevant. However, extracellular Ca^2+^ may not be the sole factor to bring the inconsistency in the role of actomyosin contractility because *in vivo* studies also showed opposing results for the effect of RhoA-ROCK-myosin II signaling perturbations on tumorigenesis in the skin (Sugai et al., 1992; Samuel et al., 2011; García-Mariscal et al., 2018). Importantly, in the previous *in vivo* studies, actomyosin activity was perturbed by pharmacologically or genetically altering expression/activity of RhoA or ROCK (Sugai et al., 1992; Samuel et al., 2011; García-Mariscal et al., 2018). Since actomyosin is not a sole downstream target of RhoA and ROCK (Riento and Ridley, 2003; Wheeler and Ridley, 2004), these previous approaches may not directly address the role of actomyosin contractility.

Another major limitation in the previous *in vivo* studies is the lack of methodology for evaluating and manipulating the actomyosin-mediated mechanical status in specific subcellular compartments such as cell-ECM and cell-cell adhesion sites. Indeed, tension at FAs and at AJs reportedly have opposing effects on cell proliferation; while tension at FAs promotes proliferation of various cell types (Schwartz and Assoian, 2001; Wang et al., 2005) and inhibits differentiation in normal keratinocytes (Carley et al., 2021), tension at AJs has been suggested to inhibit proliferation of MDCK epithelial cells (Furukawa et al., 2017), keratinocytes (Hirata et al., 2017) and cSCC cells (this study). These opposing effects may counteract each other when the actomyosin activity is globally altered in the skin tissue. Fibrillogenesis and stiffening of ECM induced by tissue-scale stretching of the skin (Verhaegen et al., 2012) may also activate integrin signaling at FAs and counter the anti-proliferative effect of AJ tension. In contrast to the former studies, we have discriminated, in the present study, the role of tension at AJs from that at FAs by combing pharmacological modulation of actomyosin activity, mechanical manipulation of cells, modulation of the cell density, pharmacological inhibition of FA signaling, and genetical perturbation of AJs. With these approaches, we have revealed that tensile loads at AJs have a specific role in inhibiting cSCC cell proliferation.

Considering the well-known proliferation-promoting role of tensile loads at FAs, it might be surprising that RhoA activation in sparse A431 cell culture did not promote cell proliferation. In contrast to RhoA-induced vinculin accumulation at AJs (Figure 3A), activation of RhoA in A431 cells did not apparently promote the recruitment of vinculin and zyxin to FAs under both confluent (Figure 3A, Supplementary Figure S2A) and sparse (Supplementary Figure S2C) conditions. These results are consistent with the previous reports that myosin II shows polarized distribution along the apicobasal axis in epithelial cells (Levayer and Lecuit, 2012), which was also observed in the present study (Figure 2C). Furthermore, MLC phosphorylation in FA-associated actomyosin stress fibers is differentially regulated by ROCK and MLCK depending on cell types (Totsukawa et al., 2004; Kassianidou et al., 2017; Matsui and Deguchi, 2019). Thus, activation of the RhoA-ROCK pathway in sparse A431 cells might not effectively increase tension at FAs and, therefore, failed to promote cell proliferation.

Since tensile loads at AJs are suggested to have an anti-malignant effect on cSCC cells through inhibition of their proliferation and promotion of their epidermal differentiation, the tensile status of AJs may provide a novel therapeutic target for cSCC. As mentioned above, global activation of actomyosin in the tissue might increase tension not only at AJs but also at FAs, which may cause undesired stimulation of cSCC growth (Sugai et al., 1992; Samuel et al., 2011; Zhou et al., 2015; Ichijo et al., 2017; Purnell et al., 2018). Thus, the development of a methodology for locally increasing tension at AJs would be required for the practical application and RhoA signaling might be a potential target. Furthermore, understanding of the molecular mechanism for attenuating malignant behaviors (hyperproliferation and poor differentiation) of cSCC cells downstream of tensile loads at AJs would also be needed for developing an effective strategy for the treatment of cSCC.

## Methods

### Mice

Wild-type FVB/N mice were purchased from CLEA Japan, Inc. (Tokyo, Japan). FVB/N mice display a high incidence of cSCC after treatment with various tumor-induction protocols (Hennings et al., 1993). All mice were housed in the animal facilities of Nagoya University Graduate School of Medicine. All animal protocols were approved by the Animal Care and Use Committee of Nagoya University Graduate School of Medicine (Approval ID number 28175).

### Cell Culture

The human cSCC cells A431 (purchased from ATCC, CRL-1555), C2C12 mouse myoblasts, and HEK293T cells were maintained in high-glucose Dulbecco’s modified Eagle’s medium (DMEM; Nacalai Tesque, Kyoto, Japan) supplemented with 10% fetal bovine serum (FBS; Life Technologies, Carlsbad, CA). The mouse primary epidermal keratinocytes (MPEK) were isolated as previously described (Sunagawa et al., 2016) and maintained in the KGM-Gold medium (Lonza, Walkersville, MD).

### Antibodies and Chemicals

The rabbit polyclonal antibodies (pAbs) against Tyr925-phosphorylated FAK (3284), MLC (3672), Ser19-phosphorylated MLC (3671) and Thr18,Ser19-doublephosphorylated MLC (3674), the rabbit monoclonal antibody (mAb) against E-cadherin (3195), GAPDH (2118), and the mouse mAb against Ser19-phosphorylated MLC (3675) were purchased from Cell Signaling Technology (Danvers, MA). The mouse mAb against keratin-10 (MA5-13705) was from Thermo Fisher Scientific (Waltham, MA). The rabbit pAbs against β-catenin (ab6302) and non-muscle myosin IIA (ab75590), and the rabbit mAb against keratin-5 (ab52635), FAK (ab40794), Tyr397-phosphorylated FAK (ab81298), and Ki-67 (SP6) were from Abcam (Cambridge, United Kingdom). The mouse mAb against β-actin (A5441) and vinculin (V9131), and the rabbit mAb against zyxin (ZRB1408) were from Sigma Chemical (St. Louis, MO). Alexa Fluor 488-goat anti-rabbit IgG, Alexa Fluor 488-goat anti-mouse IgG, Alexa Fluor 546-goat anti-rabbit IgG and Alexa Fluor 555-goat anti-mouse IgG antibodies were from Life Technologies. Horseradish peroxidase-conjugated anti-mouse IgG and anti-rabbit IgG antibodies were from GE Healthcare (Little Chalfont, United Kingdom) and Zymed Laboratories (San Francisco, CA), respectively. Rho activator II (RhoA activator) was from Cytoskeleton (Denver, CO). Para-aminoblebbistatin (Blebb) was from Optopharma (Budapest, Hungary). The FAK inhibitor PF-573228 was from Sigma Chemical. Ethylene glycol-bis (β-aminoethyl ether)-N,N,N′,N′-tetraacetic acid (EGTA) was from Dojindo Laboratories (Kumamoto, Japan). Hoechst 33342 was from Life Technologies.

### Skin Tumorigenesis Assay

Murine skin tumors were induced by a standard two-stage chemical carcinogenesis protocol, as previously described (Abel et al., 2009; Aoi et al., 2014). In brief, 6–8-week-old female mice were shaved on the dorsal skin with electric clippers and subjected to a single topical application of 100 nmol 9,10-dimethyl-1,2-benzanthracene (DMBA, Sigma Chemical) in 200 μl acetone 24 h after shaving. One week after DMBA treatment, the mice received topical applications of 5 nmol tetradecanoyl-phorbol acetate (TPA, Sigma Chemical) in 200 μl acetone twice weekly for 15 weeks. All animal protocols were approved by the Animal Care and Use Committee of Nagoya University Graduate School of Medicine (Approval ID number: 28175).

### Immunohistochemistry

Skin and tumor tissues were fixed for 24 h in 10% neutral-buffered formalin (Nacalai Tesque), dehydrated, and embedded in paraffin. Sections (4-μm thick) prepared for IHC were deparaffinized in xylene and rehydrated in a graded series of ethanol. For antigen retrieval, the sections were immersed in either Epitope Retrieval Solution (pH 6, Leica Biosystems) or HistoVT One solution (pH 7, Nacalai Tesque) and incubated for 30 min at 98°C in a water bath. Non-specific binding was blocked with Protein Block Serum-Free (Dako) for 30 min at room temperature. Then, the sections were incubated with primary antibodies diluted in 1% BSA/PBS for 2 h at room temperature. Primary antibody dilutions were as follows: anti-pS19-MLC–1:100, anti-Ki-67–1:100. After inhibiting endogenous peroxidase with 0.3% hydrogen peroxide in methanol for 15 min, the sections were further incubated with EnVision+ system-horseradish peroxidase (HRP)-Labelled Polymer (anti-rabbit, Dako) for 30 min at room temperature. Signals were visualized by the Liquid DAB+ Substrate-Chromogen System (Dako) with nuclear counterstaining using hematoxylin. Skeletal and smooth muscle cells from the regions underlying epidermis were used as an internal positive control for pS19-MLC staining (Supplementary Figures S1A,B).

### shRNA-Mediated Depletion of α-Catenin

shRNAs were introduced into A431 cells using the retrovirus system, as described previously (Hirata et al., 2017). In brief, the sequence of either 5′-GAC​TTA​GGA​ATC​CAG​TAT​A-3′ (for targeting human catenin alpha-1) or 5′-ATA​GTC​ACA​GAC​ATT​AGG​T-3′ (for the non-targeting control) was inserted into the pSUPER.retro.puro retroviral vector. The inserted vector was co-transfected with the pE-ampho vector into HEK293T cells using the GeneJuice transfection reagent (Merck Millipore, MA, United States). Supernatants containing viral particles were collected 48 h after transfection, filtered through 0.45-μm syringe filters, and used for infection into A431 cells in the presence of 8 μg/ml Polybrene (Sigma Chemical). Infected A431 cells were selected with 1.5 μg/ml puromycin (Sigma Chemical).

### Pharmacological Treatment and EdU Incorporation

Trypsinized cells were seeded at the cell density of 1 × 10^4^ cells/cm^2^ (sparse), 1 × 10^5^ cells/cm^2^ (sub-confluent), or 2 × 10^5^ cells/cm^2^ (confluent) onto glass-bottom or plastic 24-well plates, or elastic polydimethylsiloxane (PDMS) chambers (Strex, Osaka, Japan) precoated with 50 μg/ml collagen (Koken, Tokyo, Japan), and cultured for 40 h before the treatment. When cells were seeded at the confluent cell density (i.e., 2 × 10^5^ cells/cm^2^), surfaces of the wells/chambers were completely covered with cells. In sparse cultures, cells forming clusters were excluded from analyses, unless otherwise stated.

In the experiment to compare the proliferation of A431 and MPEK cells, we used the KGM-Gold medium, which promotes the proliferation of primary keratinocytes in serum-free and feeder-free conditions (Borowiec et al., 2013). For the experiments with the low Ca^2+^-medium, Ca^2+^-free DMEM (Nacalai Tesque) supplemented with 0.3 mM EGTA and 10% FBS was used (Hirata et al., 2020). Given the concentration of Ca^2+^ in FBS to be 3.4 mM (Gstraunthaler and Lindl, 2013), the free Ca^2+^ concentration in the low Ca^2+^-medium was estimated as ∼40 μM (calculated using Ca-EGTA Calculator v1.3; Bers et al., 2010). In all other experiments, DMEM supplemented with 10% FBS was used.

For the experiments with pharmacological activation/inhibition of actomyosin contractility, cells were treated with either 5 μg/ml of the membrane-permeable form of cytotoxic necrotizing factor-1 from *Escherichia coli* (hereafter RhoA activator), which deamidates glutamine-63 of RhoA to glutamate to constitutively activate RhoA by blocking its GTPase activity (Flatau et al., 1997; Schmidt et al., 1997), 100 μM of the myosin II ATPase inhibitor para-aminoblebbistatin [hereafter Blebb-a water-soluble form of blebbistatin (Straight et al., 2003; Kovács et al., 2004; Várkuti et al., 2016)], or combination of 5 μg/ml RhoA activator and 100 μM Blebb for 6 h or 24 h, followed by further incubation for 2 h with 10 μM EdU in the presence of the same set of the compounds. When indicated, along with the RhoA activator and/or Blebb, 10 μM of the FAK inhibitor PF-573228 (Slack-Davis et al., 2007) was added to the media.

For the experiments with mechanical stretching, cells seeded on the PDMS substrate were subjected to 20% uniaxial planar stretch continuously for 6 h and further incubated for 2 h with 10 μM EdU under the stretch. To stretch cells under inhibition of FAK activity, cells were pre-treated with 10 μM of the FAK inhibitor PF-573228 for 2 h and then stretched in the presence of PF-573228.

After the incubation with EdU, cells were fixed and permeabilized with 4% formaldehyde and 0.5% Triton X-100, respectively, in PBS, and incorporated EdU was visualized with Alexa Fluor 488-azide or Alexa Fluor 647-azide using the “click chemistry” kit (Life Technologies or Click Chemistry Tool, Scottsdale, AZ). Total nuclei were stained with 5 μg/ml Hoechst 33342.

### Immunofluorescence

Cells, which were pharmacologically treated when indicated, were fixed and permeabilized for 30 min with 4% formaldehyde and 0.2% Triton X-100 in the cytoskeleton stabilizing buffer (137 mM NaCl, 5 mM KCl, 1.1 mM Na_2_HPO_4_, 0.4 mM KH_2_PO_4_, 4 mM NaHCO_3_, 2 mM MgCl_2_, 5.5 mM glucose, 2 mM EGTA, and 5 mM PIPES, pH 6.1). This was followed by blocking with 1% BSA in the cytoskeleton stabilizing buffer for 30 min. The cells were then incubated with primary antibodies overnight, washed, and further incubated with secondary antibodies for 40 min. Primary antibodies were diluted in the cytoskeleton stabilizing buffer containing 1% BSA as follows: anti-keratin-5–1:250, anti-keratin-10–1:50, anti-E-cadherin–1:200, anti-vinculin–1:200, anti-zyxin–1:1000, anti-non-muscle Myosin IIA–1:250, pS19-MLC–1:250, and anti-β-catenin–1:3000. Secondary antibodies and Alexa Fluor 647-conjugated phalloidin (to detect F-actin; Thermo Fisher Scientific) were diluted to 1:200 in the cytoskeleton stabilizing buffer containing 1% BSA. Total nuclei were stained with 5 μg/ml Hoechst 33342. All immunofluorescence experiments were repeated at least twice, and typical images are shown in figures.

### Microscope Image Acquisition

For fluorescence imaging, cells were observed using an epifluorescence inverted microscope (BZ-X710, Keyence, Osaka, Japan) equipped with an air (NA 0.45, 20x or NA 0.50, 40x ELWD S Plan Fluor, Nikon) or an oil immersion (NA 1.40, 60x, Plan Apo, Nikon) objective and a high-resolution 2.83-megapixel monochrome CCD camera. The BZ-X viewer software (version 1.3.0.5, Keyence) was used for image acquisition. To characterize the adherens junctions, cells were observed using a confocal laser scanning microscope (LSM 510, Zeiss, Oberkochen, Germany) equipped with an oil immersion objective (NA 1.4, 63x Plan-Aphochromat, Zeiss) under control of ZEN 2009 software (version 6.0.0.303, Zeiss).

### Image Processing and Analysis

Acquired images were analyzed offline using custom pipelines in ImageJ (ver. 1.53c) and in the automated image analysis software CellProfiler (ver. 4.0.6, Carpenter Lab, Broad Institute of Harvard and MIT, Cambridge, MA; (McQuin et al., 2018). Briefly, images were subjected to nuclei segmentation using the StarDist 2D plugin (Schmidt et al., 2018) for ImageJ based on the Hoechst channel with the versatile (fluorescent nuclei) model. The segmented images were saved and used as an input in CellProfiler. Whenever necessary, whole cells were segmented with the propagation algorithm and the minimum cross-entropy thresholding method in the appropriate secondary staining channel by using nuclei as “seed” objects. Next, the fluorescence intensities of segmented objects were calculated. To identify the region of the cell perimeter that was in direct contact with neighboring cells, the cytoplasm surrounding the segmented nucleus was detected in the β-catenin channel. Then the adjacent objects were identified. For quantification of continuous vinculin staining, the continuous lines of junctional vinculin were manually outlined and their lengths were measured using ImageJ.

### Western Blot

Cells, which were pharmacologically treated when indicated, were lysed with the 2x lithium dodecyl sulfate sample buffer (Life Technologies) containing 2.5% β-mercaptoethanol. The lysate samples were resolved by SDS-PAGE (4–12% Bis-Tris or 3–8% Tris-Acetate gels; Life Technologies), electroblotted onto a polyvinylidene fluoride membrane (Immobilon-P, Millipore, MA, United States), blocked with 1% skim milk in TBS-T (20 mM Tris, 150 mM NaCl, 0.1% Tween-20, pH 7.5), and probed with antibodies. Primary antibodies dilutions were as follows: anti-MLC–1:800, anti-S19-MLC–1:800, anti-T18,S19- MLC–1:800, anti-K5–1:25000, anti-K10–1:2000, anti-α-catenin–1:10000, and anti-β-actin–1:10000 in 0.1% skim milk in TBS-T. Secondary HRP-conjugated antibodies were diluted to 1:2500 in 0.1% skim milk in TBS-T. Immuno-reactive bands were detected with the SuperSignal West Femto ECL substrate (Thermo Fisher Scientific). Images were captured using CoolSNAP *fx* CCD camera (Photometrics, AZ, United States) and MetaMorph software (ver. 7.5.0.0, Molecular Devices, CA, United States). Chemiluminescence was quantified using ImageJ (ver. 1.52i; Schindelin et al., 2012). All immunoblot experiments were repeated at least twice, and typical blots are shown in figures.

### Statistical Analysis

Statistical analyses were conducted using the free software for statistical computing and graphics R (version 4.0.4; R Core Team, 2019), and graphs were produced using the packages ggplot2 (Wickham, 2016) and ggsignif (Ahlmann-Eltze and Patil, 2021).

Even though the same treatment reproducibly caused a similar effect on the cell proliferation (i.e., inhibited or promoted proliferation) against the control in each experiment, the high variability in the proliferation level of the control cells led to large SD values among experiments. Thus, to unmask the actual effect of a treatment, the ratio of EdU-positive cells under the treatment was normalized by that under the control condition in each experiment. The normalization allowed us to remove the variability in the proliferation of control cells from the analysis and isolate the effect of the treatments.

The bivariate logistic regression analysis was performed to assess the association between the cell-cell contact area and the probability of entry into the S-phase. The percentage of the cell perimeter that was in direct contact with neighboring cells was used as the independent variable. The fluorescence intensity of EdU, used as the dependent variable, was dichotomized into the outcomes “1” for EdU-positive cells or “0” for EdU-negative cells. Results of model fitting were shown graphically on the plots with corresponding *p*-values.

Bar graphs were presented as mean ± SD. In box-and-whisker plots, the line, the box, and the whiskers represent the median, the interquartile range, and the tenth and ninetieth percentiles, respectively. In the EdU incorporation assay and quantitative immunofluorescence (IF), five or more fields of view in each of two or more independent experiments were analyzed for each condition with a total of more than 800 cells (sparse) or more than 10^4^ cells (confluent) per condition in each experiment was analyzed. Statistical significance was assessed using Student’s two-tailed, unpaired t-test.

## Data Availability

The original contributions presented in the study are included in the article/Supplementary Material, further inquiries can be directed to the corresponding authors.
