# Supplementary material for: Anti-Malignant Effect of Tensile Loading to Adherens Junctions in Cutaneous Squamous Cell Carcinoma Cells
Source: Front Cell Dev Biol. 2021 Nov 11;9:728383. doi: 10.3389/fcell.2021.728383 (PMC8632149; doi:10.3389/fcell.2021.728383)
Supplement: Supplementary file 1 [file DataSheet1.docx]

***Supplementary Material***

# Supplementary Figures


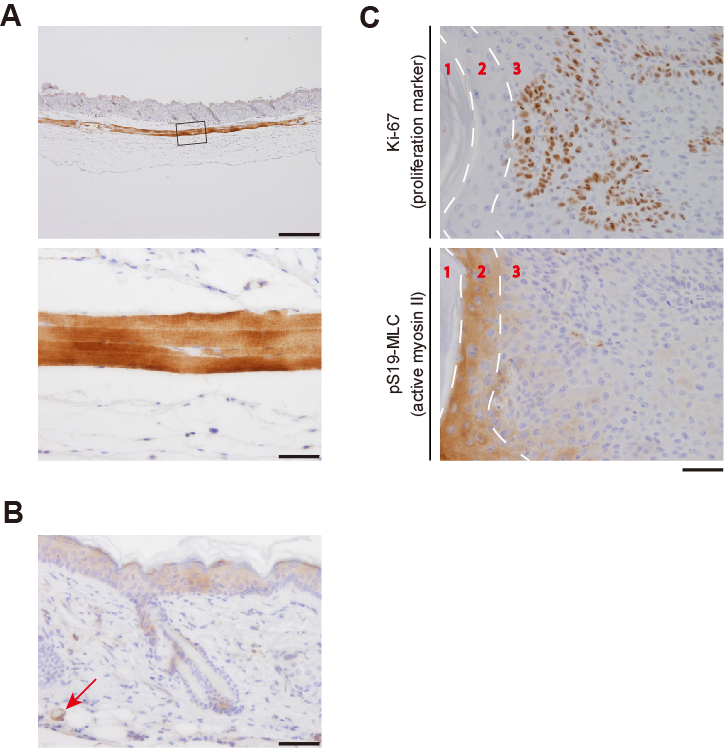


**Figure S1. IHC staining of DMBA/TPA-induced papilloma and normal back skin.**

(**A**) Normal back skin was subjected to IHC staining for pS19-MLC. A magnified image of the boxed region is also shown (bottom panel). Skeletal muscle showed high staining intensity and striated pattern indicating pS19-MLC antibody specificity. Scale bars, 500 μm for large-field image (upper panel), and 50 μm for magnified image (bottom panel).

(**B**) Normal back skin was subjected to IHC staining for pS19-MLC. Smooth muscle cells (red arrow) showed high staining intensity indicating pS19-MLC antibody specificity. Scale bar, 50 μm.

(**C**) DMBA/TPA-induced papilloma were subjected to IHC staining for Ki-67 and pS19-MLC. Stratum corneum (1), superficial (2), and deeper (3) keratinocytes layers are demarked. Scale bar, 50 μm.


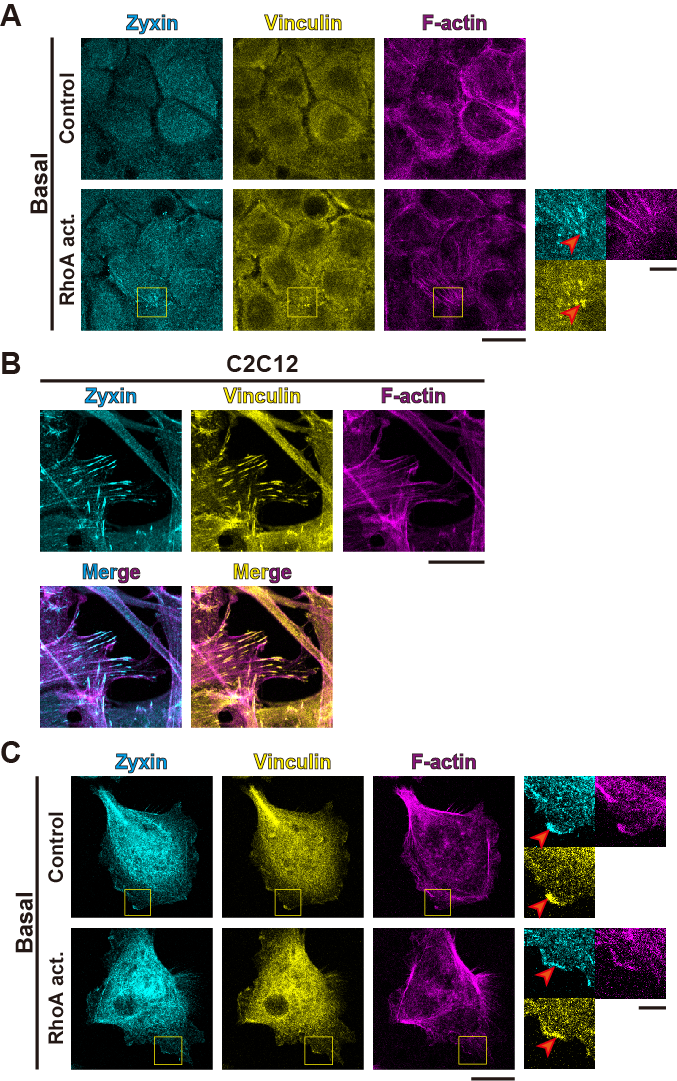


**Figure S2. Vinculin and zyxin accumulation at FAs in A431 cells is not promoted by RhoA activation.**

(**A**) Confluent A431 cells were treated with or without 5 μg/mL RhoA activator (RhoA act.) and stained for F-actin (magenta), zyxin (cyan), and vinculin (yellow). The basal focal planes of the cells are shown. Magnified images of the boxed region are also shown. The red arrowheads indicate faint FAs. Scale bars, 5 μm for magnified images, and 20 μm for others.

(**B**) Non-treated C2C12 cells were stained for F-actin (magenta), zyxin (cyan), and vinculin (yellow). The basal focal planes of the cells are shown. Scale bars, 20 μm.

(**C**) Sparse A431 cells were treated with or without 5 μg/mL RhoA activator (RhoA act.) and stained for F-actin (magenta), zyxin (cyan), and vinculin (yellow). The basal focal planes of the cells are shown. Magnified images of the boxed regions are also shown. The red arrowheads indicate accumulation of zyxin and vinculin at FAs. Scale bars, 5 μm for magnified images, and 20 μm for others.


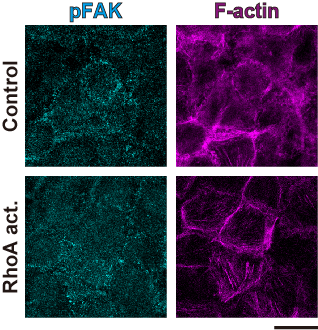


**Figure S3. FAK phosphorylation is not altered by RhoA activator treatment in confluent A431 cells.**

Confluent A431 cells were treated with or without 5 μg/mL RhoA activator (RhoA act.) and stained for F-actin (magenta) and pY925-FAK (cyan). The basal focal planes of the cells are shown. Scale bar, 20 μm.


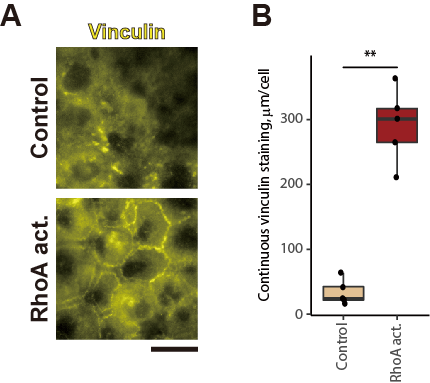


**Figure S4. RhoA activation induces junctional vinculin enrichment in cSCC cells.**

(**A**) Confluent A431 cells were treated with or without 5 μg/mL RhoA activator (RhoA act.) and stained for vinculin. Scale bar, 20 μm.

(**B**) Quantification of the continuous vinculin staining measured in confluent A431 cells treated with or without 5 μg/mL RhoA activator (RhoA act.). Length of continuous vinculin staining was measured in each image frame and normalized with the number of cells in the frame. Box-and-whisker plots show the median, the interquartile range, and the tenth and ninetieth percentiles. n = 5 fields of view from one independent experiment. **p < 0.01. Scale bar 20 μm.


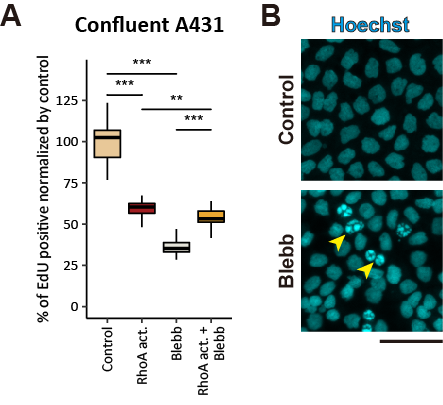


**Figure S5. Effect of the long-term actomyosin perturbations in confluent A431 cells.**

(**A**) Ratios of EdU-positive nuclei in confluent A431 cells treated with 5 μg/mL RhoA activator (RhoA act.), 100 μM p-aminoblebbistatin (Blebb) or combination of both drugs (RhoA act. + Blebb) for 24 h followed by incubation with EdU for 2 h in the presence of the same compounds. Values were normalized with the mean value in control cells. Box-and-whisker plots show the median, the interquartile range, and the tenth and ninetieth percentiles. n = 20 fields of view from one independent experiment. **p < 0.01; ***p < 0.001.

(**B**) Confluent A431 cells were treated with or without 100 μM p-aminoblebbistatin (Blebb) for 24 h and the nuclei were stained with Hoechst 33342. Yellow arrowheads show fragmented nuclei as indicators of apoptosis. Scale bar, 50 μm.


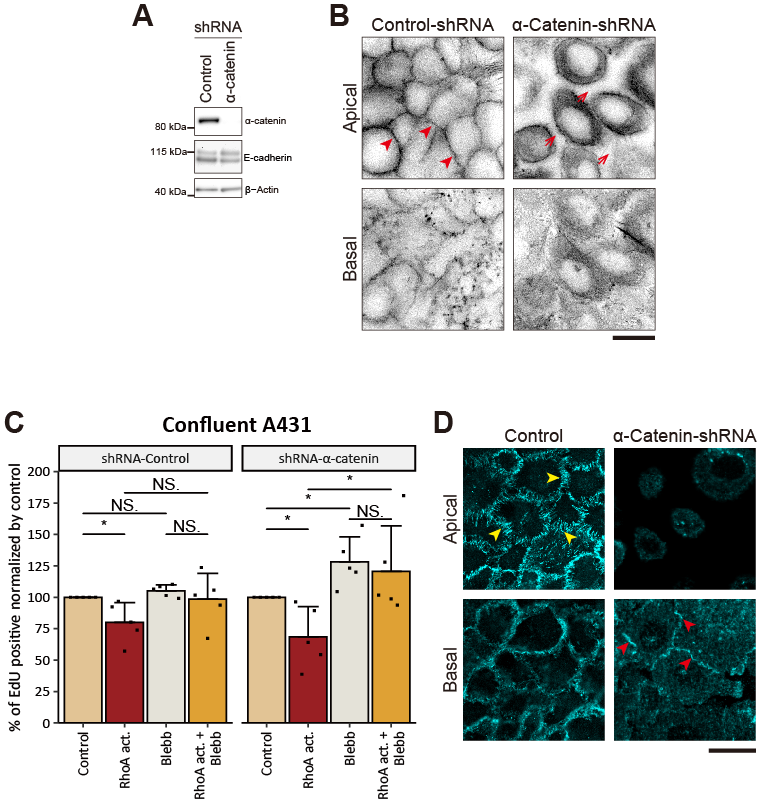


**Figure S6. Effect of α-catenin knockdown on the RhoA-induced inhibition of A431 cell proliferation.**

(**A**) Whole-cell lysates of confluent A431 cells expressing α-catenin-shRNA or control-shRNA were immunoblotted for α-catenin, E-cadherin, and β-actin.

(**B**) Confluent A431 cells expressing α-catenin-shRNA or control-shRNA were stained for F-actin. Apical and basal focal planes of the cells are shown. At the apical part, cell-cell contacts were formed in A431 cells expressing control-shRNA (red arrowheads), but not in A431 cells expressing α-catenin-shRNA (red arrows). Scale bar, 25 μm.

(**C**) Ratios of EdU-positive nuclei in confluent A431 cells expressing control-shRNA or α-catenin-shRNA treated with or without 5 μg/mL RhoA activator (RhoA act.), 100 μM p-aminoblebbistatin (Blebb) or combination of both drugs (RhoA act. + Blebb) for 6 h followed by incubation with EdU for 2 h in the presence of the same compounds. Values were normalized with the mean values in control cells. Each bar represents mean ± SD. n = 5 independent experiments. *p < 0.05; NS, p > 0.05.

(**D**) Confluent A431 cells expressing α-catenin-shRNA were stained for β-catenin as a marker of AJs. The apical and basal focal planes of the cells are shown. Red arrowheads indicate the accumulation of β-catenin along the cell-cell boundaries at the basal focal plane. Yellow arrowheads indicate mature zipper-like AJs at apical focal plane. Scale bar, 25 μm.


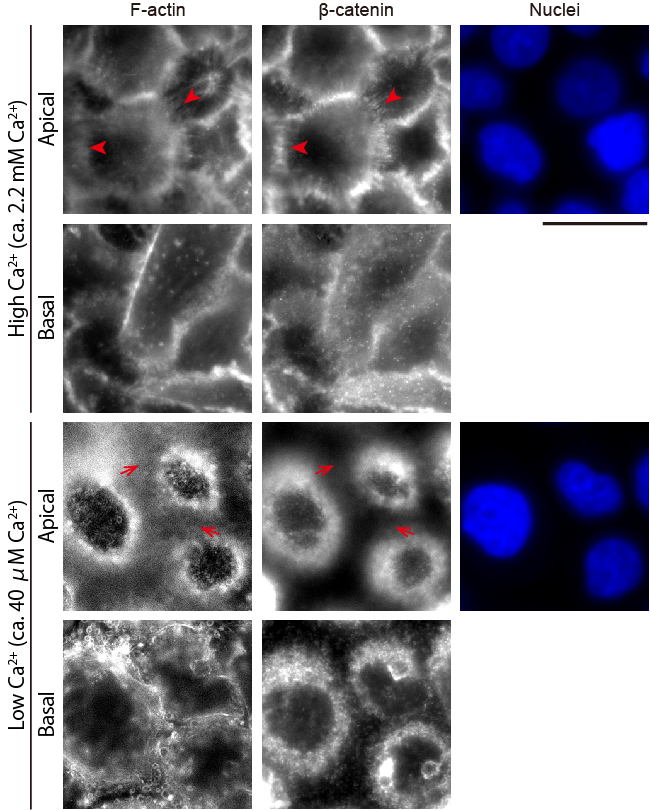


**Figure S7. Organization of the actin cytoskeleton and AJs in confluent A431 cells at different Ca2+ conditions.**

Confluent A431 cells cultured in high-Ca2+ (ca. 2.2 mM Ca2+) and low-Ca2+ (ca. 40 μM Ca2+) media were stained for F-actin and β-catenin. Apical and basal focal planes of the cells are shown. At the apical part, A431 cells formed cell-cell contacts in the high-Ca2+ medium (red arrowheads), but not in the low-Ca2+ medium (red arrows). Cell nuclei were labeled with Hoechst 33342 (blue). Scale bar, 25 μm.
